# Psychological aspects of sexuality in adults with social anxiety disorder

**DOI:** 10.1186/s12888-026-08138-2

**Published:** 2026-05-05

**Authors:** Christina Elling-Lueder, Man-Long Chung, Andreas J. Forstner, Carsten Spitzer, Stefanie Rambau, Markus Ramm, Franziska Geiser, Johannes Schumacher, Nicole Ernstmann, Katja Brenk-Franz, Rupert Conrad

**Affiliations:** 1 Department of Psychosomatic Medicine and Psychotherapy, University Hospital Muenster, Albert-Schweitzer-Campus 1, 48149 Muenster, Germany; 2 Institute of Human Genetics, University Hospital Bonn, Venusberg-Campus 1, 53127 Bonn, Germany; 3 Institute of Neuroscience and Medicine (INM-1), Research Centre Juelich, Wilhelm-Johnen-Straße, 52428 Juelich, Germany; 4 Department of Psychosomatic Medicine and Psychotherapy, University Medical Center Rostock, Gehlsheimer Straße 20, 18147 Rostock, Germany; 5 Department of Psychosomatic Medicine and Psychotherapy, University Hospital Bonn, Venusberg-Campus 1, 53127 Bonn, Germany; 6 Centre for Human Genetics, University of Marburg, Baldingerstraße, 35033 Marburg, Germany; 7 Department of Psychosomatic Medicine and Psychotherapy, Center for Health Communication and Health Services Research (CHSR), University Hospital Bonn, Venusberg-Campus 1, 53127 Bonn, Germany; 8 Chair of Health Services Research, Institute of Medical Sociology, Health Services Research, and Rehabilitation Science, Faculty of Medicine, University Hospital Cologne, University of Cologne, Cologne, Germany; 9 Institute of Psychosocial Medicine, Psychotherapy and Psychooncology, University Hospital Jena, Stoystrasse 3, 07740 Jena, Germany

**Keywords:** Social anxiety disorder, Sexuality, Sexual behavior, Gender

## Abstract

**Background:**

Sexual dysfunctions in individuals with social anxiety disorder (SAD) have been previously reported. However, most of these results refer to physical and behavioral measures. Psychological aspects have not been previously researched.

**Method:**

In the present study, we utilized an online version of the “Multidimensional Sexuality Questionnaire” (MSQ) in a sample of individuals with SAD (*n* = 242, 40.70 ± 13.40 years, 58.7% female). We hypothesized greater difficulties for SAD individuals compared to controls without SAD through the influence of fear and avoidance symptoms.

**Results:**

Based on multivariate analyses (MANCOVA), SAD individuals showcased significant deficiencies in almost all subscales of the MSQ compared to the control group (partial η^2^ = 0.016 − 0.217, all *p* < .001). Moreover, men with SAD were significantly more preoccupied and motivated for sexual behaviour and relationships than women with SAD (partial η^2^ = 0.104 − 0.159, all *p* < .001).

**Conclusion:**

These results give first insights for psychological reasons possibly underlying sexual difficulties in SAD patients. SAD individuals spend less time thinking about and are less motivated for sexuality. Assertiveness and the belief of one’s control and autonomy of sexuality are less pronounced in SAD individuals. Those signs can be approached via different techniques and therapeutic interventions if difficulties with sexuality and sexual satisfaction are relevant for those affected by SAD.

**Clinical trial number:**

Not applicable.

## Background

Among all anxiety disorders, social anxiety disorder (SAD) has one of the lowest age of onsets, indicating a very early manifestation of socially inhibiting and fear inducing symptoms for around 7–13% of individuals worldwide [1–4]. Characterized by fear of negative evaluation and feedback from others [5], individuals with SAD face difficulties in interpersonal relationships and social situations [6].

College students with higher self-reported social anxiety evaluated their sexual encounters as less pleasurable and less connected compared to others with lower social anxiety [7]. Kashdan et al. suggest that increased social anxiety may heighten self-awareness and fear of negative evaluation, leading to avoidance behaviors in sexual situations [7]. These factors can disrupt emotional presence and mutual attunement, thereby reducing both the perceived pleasure and the sense of connectedness during sexual experiences. Similarly, Montesi et al. found that among undergraduate students in intimate and committed partnerships lasting at least three months, higher social anxiety predicted sexual dissatisfaction [8]. This effect was mediated through increased fear of intimacy and dissatisfaction with open sexual communication [8], highlighting a potential pathway through which social anxiety symptoms impact sexual satisfaction. Moreover, a recent study suggests that individuals with SAD may not experience increased positive affect following sexual activity [9].

When considering clinically diagnosed SAD, similar patterns emerge. SAD has been linked to current sexual dissatisfaction and [10] and fewer sexual thoughts compared to controls [11].

Interestingly, there seem to be biological sex and gender-specific differences regarding the impact of SAD symptoms and SAD itself on sexual behavior and satisfaction^1^. Delayed ejaculation has been reported more frequently in men with SAD compared to controls [11] while findings on premature ejaculation remain inconsistent [11–15]. Kashdan et al. reported gender-specific differences in sexual contact frequency, with lower frequencies in women with high versus low self-reported social anxiety [7]. Differences in men with SAD were reversed and smaller, e.g., men with SAD had a higher likelihood of sex than men without SAD. This partly aligns with Bodinger et al., where women with SAD reported fewer sexual thoughts and sexual desire than women without SAD while no differences were found in men [11]. Additionally, SAD women had less sexual partners than women without SAD and SAD men had more paid partners than men without SAD. Moreover, higher anxiety symptom severity was linked to lower sexual satisfaction only among females [12].

Evolutionary and biopsychosocial theories offer complementary perspectives on sex and gender differences in sexual behavior. From an evolutionary standpoint, men and women have historically followed distinct reproductive strategies with men maximizing reproductive opportunities through multiple partners, and women prioritizing partner selectivity and caregiving due to greater biological investment [16–18]. However, these tendencies are shaped and reinforced by sociocultural norms. Culturally constructed sexual scripts influence how sexuality is expressed and evaluated, often encouraging greater sexual activity in men while constraining it in women. These expectations are rooted primarily on social roles rather than purely biological differences [18, 19] and may influence not only sexual behavior but also how sexual experiences are interpreted and reported, as well as how disorders such as SAD manifest across genders. Although the present study relies on biological sex due to the structure of the available data, the findings are interpreted in light of gendered sociocultural norms and sexual scripts.

Previously studies examining the impact of SAD on sexual behavior and satisfaction have primarily focused on behavioral and physiological aspects often relying on small or highly specific samples (e.g. college students or undergraduates). Moreover, they rarely explored psychological constructs that might explain the observed sexual impairments. To address these gaps, the present study uses a psychological perspective on sexual experiences in SAD, aiming to better understand how individuals with SAD perceive and evaluate their sexuality.

SAD is characterized by fear of negative evaluation, heightened self-awareness, and avoidance of socially intimate situations [5]. These aspects are likely to extend into sexual contexts, shaping not only behavior but also cognitive and affective experiences. Given that core symptoms of SAD such as fear of negative evaluation and heightened self-awareness directly affect internal experiences, examining psychological aspects of sexuality such as sexual-esteem, sexual-assertiveness, sexual satisfaction, sexual-preoccupation, sexual-consciousness, sexual-motivation and self-monitoring is particularly important for understanding how sexual difficulties emerge and are maintained in this population- Individuals with SAD may limit opportunities for intimacy, disengage during sexual encounters or withhold sexual expression, which can reduce pleasure and connection [7]. Prior research also suggests sex- and gender-specific differences in SAD populations [7, 11], yet these have not been sufficiently examined at the level of psychological sexual constructs. By integrating evolutionary, biopsychosocial, and sexual script perspectives, this study investigates gender differences in the sexual self-concept of individuals with SAD compared to controls. This approach extends previous research by focusing on underlying cognitive, affective, and motivational dimensions rather than solely on observable behavior. Examining psychological aspects of sexuality in individuals with SAD is particularly important as difficulties in sexual functioning may not only result from SAD but also contribute to its maintenance, creating a potentially bidirectional circle. A better understanding of these psychological aspects may refine theoretical models of the association between anxiety disorders and sexual functioning, inform the development of targeted interventions, and encourage clinicians to address sexual wellbeing as part of comprehensive assessment and treatment for SAD.

Our hypotheses are as follows:

1. Individuals with SAD will report lower levels of sexual-esteem, sexual-assertiveness, and sexual-satisfaction, as well as higher levels of sexual-anxiety, fear-of-sex and self-monitoring, compared to individuals without SAD.
2. Men with SAD will report higher scores in sexual-preoccupation, sexual-consciousness, sexual-motivation, sexual-assertiveness, and self-monitoring than women with SAD.

## Methods

This study is a part of the project “Social Phobia Research”, which is a collaboration between the Departments of Psychosomatic Medicine and Psychotherapy at University Hospital Münster and University Hospital Bonn, the Institute of Human Genetics, University Hospital Bonn and in cooperation with the Centre for Human Genetics, University of Marburg.

### Sample

Recruitment of participants took place within the department of Psychosomatic Medicine and Psychotherapy in Bonn through different communication paths including own clinical services, internet advertisements, television and radio, newspaper/articles and others. Inclusion criteria were a lifetime diagnosis of SAD assessed via the Structured Clinical Interview for DSM-IV axis-I disorder (SCID-I) and minimum age of 18 years. Individuals were excluded if a comorbid psychotic disorder, insufficient German language skills or difficulties in completing the questionnaires were present.

Assessment period of this cross-sectional sub study was from 2017 to 2018. 575 SAD individuals who previously participated in “Social Phobia Research” were contacted and asked for another participation via email. An additional mail was sent eight weeks after the initial contact if no participation was registered. This process was repeated three times. Data were collected through the online assessment tool “SurveyMonkey”. All participants signed an informed consent. The procedures performed in this study were in accordance with the ethical standards of the institutional and/or national research committee and with the 1964 Helsinki Declaration and its later amendments or comparable ethical standards. This study was approved by the local ethics committee of the University of Bonn (No. 222/12).

Participants data were excluded from further analyses, if more than one item of a subscale was missing. Due to missing data, 242 out of the 271 SAD participants who took part in the study were included in the final analysis. Norm values from the control group, the German validation sample, were derived from Brenk-Franz & Strauß (2011) [20].

### Assessment

#### Demographics

Basic demographic information, e.g., age, sex, partnership status and educational level, were assessed via a self-constructed questionnaire.

#### Diagnoses

Lifetime SAD and other comorbidities were diagnosed and assessed through trained interviewers via SCID-I.

#### MSQ

The “Multidimensional Sexuality Questionnaire” [MSQ; 21] is a suitable tool to investigate psychological dimensions that might play a role in sexual difficulties of SAD individuals. An online assessment of the German version of the MSQ [20] was utilized to assess psychological tendencies of sexuality. The questionnaire consists of 61 items, where the last item gives an additional reference whether the given answers are based on a present, past or an imagined sexual relationship. Participants responded to 60 items using a 5-point Likert scale (0 = “not at all”, 4 = “strongly agree”). We handled single missing items within a subscale by replacing each missing value with the mean of the remaining items for the respective participant, defined as person-mean imputation [22].

The questionnaire assesses twelve distinct subscales, each reflecting a specific dimension of sexual self-concept: *Sexual-esteem* reflects an individual´s belief in one´s own sexual competence and capacity to relate intimately with others (e.g., “I am confident about myself as a sexual partner”). *Sexual-preoccupation* refers to a tendency to think frequently about sex (e.g., “I think about sex all the time”). *Internal-sexual control* measures the idea that individuals have control over the sexual aspects of their lives (e.g., “My sexuality is something that I am largely responsible for”). *Sexual-consciousness* captures the degree of awareness one has of their own sexual thoughts and behaviors (e.g., “I am very aware of my sexual feelings”).

*Sexual-motivation* measures the drive or desire for sexual activity (e.g., “I am very motivated to be sexually active”). *Sexual-anxiety* describes feelings of tension, worry, or discomfort regarding the sexual dimension of one´s life (e.g., “I feel anxious when I think about the sexual aspects of my life”). *Sexual-assertiveness* measures the ability to express one´s sexual needs and preferences, (e.g., “I am very assertive about the sexual aspects of my life”). *Sexual-depression* describes the tendency toward depressive feelings related to one´s sexual life (e.g., “I am depressed about the sexual aspects of my life”).

*External-sexual control* measures the idea that human sexuality is influenced by factors beyond individual´s control (e.g., “The sexual aspects of my life are determined mostly by chance happenings”). *Self-monitoring* reflects the extent to which individuals attend to how their sexual behavior may be perceived by others (e.g., “I sometimes wonder what others think of the sexual aspects of my life”). *Fear-of-sex* captures fear of participating in sexual activities with another person. (e.g., “I am somewhat afraid of becoming sexually involved with another person”), and *sexual-satisfaction* assesses perceived fulfillment and contentment with one´s sexual life (e.g., “I am very satisfied with the way my sexual needs are currently being met”).

Higher values in each of the twelve subscales represent a higher degree of the construct, accordingly. In this study, all twelve subscales demonstrated acceptable to excellent internal consistency. Cronbach´s alpha values were as follows: .89 for sexual-esteem, .91 for sexual-preoccupation, .68 for internal-sexual control, .74 for sexual-consciousness, .90 for sexual-motivation, .90 for sexual-anxiety, .88 for sexual-assertiveness, .89 for sexual-depression, .76 for external-sexual control, .76 for self-monitoring, .90 for fear-of-sex and .83 for sexual-satisfaction (all values reported prior to person-mean imputation).

### Statistical analyses

Differences in demographics (age, sex, partnership and educational level) between SAD individuals and controls were analysed via *t*-tests for continuous variables and via chi^2^-tests for categorical variables. These analyses were considered descriptive therefore no correction for multiple testing was applied. To account for the intercorrelations among MSQ subscales, group and gender differences were examined using multivariate analyses of covariance (MANCOVA), with age, partnership status, educational level and (in the SAD group) lifetime depressive disorder included as covariates. Partial η^2^ (partial eta squared) was used as a measure of effect size. Values of approximately 0.01, 0.06 and 0.14 were interpreted as indicating small, medium and large effects [23]. To account for potential effects of comorbid depressive disorder, an additional MANCOVA was conducted in the SAD group including lifetime depressive disorder (yes/no) as a factor and age, partnership status and educational level as covariates.

## Results

There were no significant differences between included and excluded participants regarding age and gender. A comparison with the control group displays significant differences in age, current partnership and educational degree. SAD individuals in our sample were older, reported fewer current partnerships and had more individuals with a high school degree and below (Table 1). After controlling for age, educational level and partnership status a MANCOVA revealed no significant differences between participants in the SAD group with and without lifetime depressive disorder on the MSQ subscales.

**Table 1:** Sample characteristics

		**Control**	**SAD**	*t / χ* ^*2*^	*p*	*d /* φ
		*n* = 261 (52%)	*n* = 242 (48%)			
		*n* (%) / mean (*SD*)	*n* (%) / mean (*SD*)			
Sociodemographics						
Gender (women)		144 (55.2%)	142 (58.7%)		ns	
age		28.75 (8.89)	40.70 (13.40)	11.86	**< .001**	1.07
current partnership (yes)		201 (77.0%)	110 (47.8%)	44.85	**< .001**	0.302
Educational degree						
Below high school		127 (48,7%)	180 (74.4%)	10.62	**.001**	0.146
High school		134 (51.3%)	154 (25.6%)			

Note SAD = social anxiety disorder, *t* = t-Value, *χ*^*2*^  = Chi-square, *p* = p-Value, *d =* Cohen’s d, φ = Cramer’s V, *n* = sample size, *SD* = standard deviation

We observed significant differences for almost all subscales of the MSQ between the SAD sample and the control group, confirming our first hypothesis. Only the subscale “external-sexual control” did not reach significance (Table 2). All other subscales showed significant differences.

**Table 2:** MSQ subscales - group comparison

	**Control**	**SAD**	*F*	df_1_	df_2_	*p*	part. η^2^
	*n* = 261 (53.7%)	*n* = 225 (46.3%)					
	mean (*SD*)	mean (*SD*)					
MSQ Scales							
1. Sexual-Esteem	13.56 (3.75)	6.84 (5.48)	132.93	1	481	**< .001**	0.217
2. Sexual-Preoccupation	6.68 (4.82)	4.50 (4.82)	22.68	1	481	**< .001**	0.045
3. Internal-Sexual Control	14.61 (3.28)	11.86 (3.89)	33.18	1	481	**< .001**	0.065
4. Sexual-Consciousness	15.43 (3.25)	11.17 (4.41)	94.34	1	481	**<.001**	0.164
5. Sexual-Motivation	12.95 (4.31)	8.71 (5.79)	43.67	1	481	**< .001**	0.083
6. Sexual-Anxiety	3.11 (3.56)	8.23 (6.10)	75.66	1	481	**<.001**	0.136
7. Sexual-Assertiveness	12.33 (4.68)	6.79 (5.36)	59.37	1	481	**<.001**	0.110
8. Sexual-Depression	3.16 (3.57)	9.15 (6.03)	91.56	1	481	**<.001**	0.160
9. External-Sexual Control	5.46 (4.59)	7.34 (4.84)	0.43	1	481	.512	0.001
10. Self-Monitoring	3.44 (4.02)	4.95 (4.65)	8.81	1	481	**.003**	0.018
11. Fear-of-sex	4.69 (3.86)	11.14 (5.94)	7.93	1	481	**.005**	0.016
12. Sexual-Satisfaction	12.36 (3.79)	7.49 (5.70)	95.80	1	481	**<.001**	0.166

Note: SAD = social anxiety disorder, *F* = F-Statistic, *part. n*^*2*^ *= partial eta squared*, df_1_ = numerator degrees of freedom, df_2_ = denumerator degrees of freedom, MSQ = multidimensional sexuality questionnaire, *n* = sample size, *SD* = standard deviation

Our second hypothesis was confirmed, as men with SAD scored higher than women with SAD in the subscales “sexual-preoccupation”, “sexual-consciousness”, “sexual-motivation”, “sexual-assertiveness” and “self-monitoring” (Table 3).

**Table 3:** SAD sub sample comparison – gender

	**SAD men**	**SAD women**	*F*	df_1_	df_2_	*p*	part. η^2^
	*n* = 91 (40.6%)	*n* = 133 (59.4%)					
	mean (*SD*)	mean (*SD*)					
MSQ Scales							
1. Sexual-Esteem	7.53 (5.57)	6.37 (5.41)	2.74	1	218	.099	0.012
2. Sexual-Preoccupation	6.86 (5.33)	2.90 (3.69)	41.29	1	218	**<.001**	0.159
3. Internal-Sexual Control	12.12 (3.54)	11.66 (4.13)	0.84	1	218	.360	0.004
4. Sexual-Consciousness	12.51 (4.06)	10.26 (4.44)	12.24	1	218	**<.001**	0.053
5. Sexual-Motivation	10.79 (5.71)	7.29 (5.44)	25.24	1	218	**<.001**	0.104
6. Sexual-Anxiety	8.30 (5.78)	8.19 (6.36)	0.05	1	218	.826	0.000
7. Sexual-Assertiveness	7.56 (5.48)	6.25 (5.25)	4.56	1	218	**.034**	0.020
8. Sexual-Depression	9.41 (5.95)	8.97 (6.12)	0.16	1	218	.689	0.001
9. External-Sexual Control	7.75 (4.45)	7.10 (5.08)	0.03	1	218	.866	0.000
10. Self-Monitoring	6.15 (4.92)	4.13 (4.29)	12.01	1	218	**<.001**	0.052
11. Fear-of-sex	10.32 (5.41)	11.64 (6.22)	3.66	1	218	.057	0.017
12. Sexual-Satisfaction	7.82 (5.89)	7.24 (5.60)	1.52	1	218	.219	0.007

Note: SAD = social anxiety disorder, *F* = F-Statistic, *part. n*^*2*^ *= partial eta squared*, df_1_ = numerator degrees of freedom, df_2_ = denumerator degrees of freedom, MSQ = multidimensional sexuality questionnaire, *n* = sample size, *SD* = standard deviation

## Discussion

This study investigates psychological dimensions underlying sexual behavior in a large, clinical and treatment-seeking SAD sample. Previous studies have shown several sexual deficiencies for those affected by SAD e.g., less pleasurable, and enjoyable intimate experiences and sexual dissatisfaction [7–11]. The results of the present study show that SAD individuals face difficulties in various dimensions as captured by the MSQ. They tend to be more afraid, dissatisfied, less assertive and have lower esteem of their sexual life compared to the control sample. Additionally, SAD individuals spend less time with sexual thoughts, their own sexuality and sexual behavior, are more depressed of their sexual life and believe their sexuality is out of their control and reach.

While self-esteem grows naturally over the course of life, SAD symptoms, compared to other anxiety symptoms, have the strongest negative impact on self-esteem development [24]. Furthermore, SAD individuals more frequently report fearful, insecure, preoccupied and avoidant attachment styles [25–27], which also has been linked to dissatisfaction in sexual life [28, 29]. Fitting these findings, the highest effect sizes were found for the subscales “sexual-esteem” and “sexual-satisfaction”, indicating that the greatest difficulties for SAD individuals are enjoying sexual activities and being confident engaging in intimate relationships.

A new insight into characteristics associated with these difficulties is provided by the subscale “internal-sexual control”, defined as “the belief that the sexual aspects of one’s life are determined by one’s own personal control” [20]. SAD individuals scored significantly lower than controls, indicating a perception of reduced control over their own sexuality. This may reflect the more passive and avoidance-oriented behavioral patterns characteristic of SAD, which also manifest in lower sexual-assertiveness. The ability to properly communicate one’s sexual needs and desires is key to overall satisfaction and well-being [30] yet fear of negative evaluation may prevent SAD individuals from expressing their needs or reflecting on their sexual lives.

The subscale “self-monitoring” also showed a significant difference between controls and SAD individuals. According to Snell et al., 1993, “self-monitoring” reflects “the tendency to be aware of the public impression which one’s sexuality makes on others” [20]. Due to the fear of negative evaluation and feedback from others, this association may reflect a greater attentional focus on perceived social evaluation rather than a causal influence on sexual behavior. Although sexual behavior is a private domain and everyday behavior, e.g. eating in front of others, are typically more salient for individuals with SAD, the SAD group showed a higher level of “self-monitoring”. This indicates that concerns about public evaluation may extend even to intimate aspects of sexuality.

Gender differences were also observed. In particular, “self-monitoring” appears to be more salient for men with SAD, who reported higher “self-monitoring” scores than women with SAD. These differences can be interpreted in light of theoretical frameworks that consider evolutionary and biopsychosocial factors in gender-specific sexual behavior. For instance, evolutionary theories suggest that men may be more focused on sexual opportunities and their own sexual presentation [31], while sociocultural aspects may emphasize male sexual competence and desirability [18, 19]. Psychologically, higher levels of “self-monitoring” in men with SAD may reflect increased attention to social evaluation in sexual contacts. Against this background, it is not surprising that our second hypothesis was supported. Men with SAD tend to spend more time reflecting on their own sexuality, reported a higher desire to be in a sexual relationship and showed greater awareness of how others might perceive their sexual behavior compared to women with SAD. Even in a specific group of people, where intimacy and close relationships can be potential stressors, men appeared more inclined to engage in sexual relationships than women, although on a descriptive level, it is still below controls [21].

Although these results provide informative associations, the cross-sectional nature of our study precludes any causal interpretation. This limitation means that alternative explanations, such as sexual dissatisfaction contributing to the maintenance or exacerbation of social anxiety, cannot be ruled out. Additionally, comorbid depressive disorder may contribute to these associations, as it was only controlled for in the SAD group and conceptually overlaps with several MSQ subscales [32]. In line with attachment theory, individuals high in attachment avoidance tend to withdraw from close relationships [33] which may extend to reduced engagement in intimate contexts, consistent with our observed associations. Furthermore, cognitive-behavioral models of social anxiety emphasize increased self-focused attention and monitoring in socially evaluative contexts [34] which may both result from and contribute to sexual difficulties. Taken together, these frameworks suggest potential bidirectional relationships between SAD and sexual functioning. Therefore, longitudinal research is needed to clarify the directionality of these relationships.

Differentiating the hampering effects of SAD symptoms from the evolutionarily and biopsychosocially rooted aspects of sexuality in men may provide a useful framework for considering potential therapeutic approaches. The present findings suggest that individuals with SAD report less engagement with their sexuality, including reduced time spent thinking about, engaging in and satisfying sexual needs. However, these associations should not be interpreted as correlational rather than causal and the possibility of bidirectional or reverse effects must be acknowledged. From a clinical perspective, these associations may point to potentially relevant therapeutic interventions. For example, different approaches such as working on communicating own sexual needs and desires (self-disclosure) may be beneficial. In a sample of heterosexual couples, one own’s self-disclosure was associated with one own’s sexual satisfaction after controlling for partnerships [35]. Working on assertiveness may be relevant for all genders and generally beneficial for those affected by SAD as it reduces SAD symptoms [36]. Since the MSQ subscales are referring to different but explicit dimensions of sexuality, interventions and techniques could be derived and tailored according to each subscale. In particular, cognitive-behavioral techniques may help to address maladaptive beliefs related to internal- and external-sexual control, such as perceived lack of internal control or the overvaluation of external control, similarly to those observed in depressive symptomatology. Exposure-based interventions adapted to intimate and relationship contexts may help to reduce anxiety related to sexual interactions and perceived evaluation. Moreover, psychoeducation may normalize sexual concerns and reduce shame associated with sexuality in individuals with SAD. Additionally, mindfulness and body-focused interventions may be useful to reduce excessive self-focused attention.

### Limitations

While highlighting novel aspects of sexuality in a large sample of people with SAD, the present study has some limitations. First, the cross-sectional design does not allow for causal interpretations and all findings should be interpreted as correlational. Second, although the MSQ gives important insight into psychological aspects of sexuality, it is a self-report questionnaire which is prone to social desirability, especially with such an intimate and personal topic like sexuality. Furthermore, the control group was not part of our data acquisition, resulting in differences in assessment conditions. An additional limitation concerns the temporal mismatch between the clinical SAD sample (2017–2018) and the normative control data (2011). Potential socio-cultural changes over time may have influenced the results and it remains unclear whether group differences reflect disorder-specific effects or broader societal changes. Additionally, measurement invariance across time was not tested and therefore it cannot be ensured that the constructs were assessed equivalently in both groups. Although a binary measure of lifetime depressive disorder was included in additional analyses in the SAD group, the lack of assessment of depressive symptoms in the control group limits the ability to rule out residual confounding. Therefore, the findings should be interpreted with caution. Nonetheless, it is one of the first studies addressing this important subject in a large clinical SAD sample.

### Future research

Future research should aim to address these limitations by using longitudinal or experimental designs to allow for causal inferences. Including well-matched control groups assessed under identical conditions would further strengthen the validity of comparisons. In addition, future studies should assess depressive symptoms and other relevant comorbidities to better disentangle disorder-specific effects from broader internalizing processes. Finally, future research using factor-analytic designs could further clarify whether the observed MSQ structure reflects distinct psychological constructs or a generalized sexuality-related distress factor.

## Conclusion

This study gives novel insights to psychological aspects of sexuality and sexual behavior in individuals with SAD. Compared to a control group, SAD individuals show more deficiencies in a variety of sexual behavior as captured by the MSQ. Gender-specific differences were present, too. However, they seemingly do not have any influence on overall sexual satisfaction and sexual esteem. Different therapeutic approaches can be derived and utilized to further increase sexual satisfaction when needed.

## Data Availability

The authors have not deposited the data in a publicly accessible data base.

## Footnotes

1 This study examines sex and gender within a binary framework, reflecting the structure of the available data, while acknowledging the existence and significance of non-binary and diverse gender identities. In this paper, *sex* refers to the biological and physiological characteristics that distinguish males and females, such as chromosomes, hormones, and reproductive anatomy. *Gender*, on the other hand, refers to the socially and culturally constructed roles, norms, and identities associated with being male, female, or another gender.
